# A Red-Emitting COF Ionic Exchanged With Green-Emitting Tb(III) Complex Anion: Synthesis, Characterization, Ratiometric Emission Sensing, and Removal of Picric Acid

**DOI:** 10.3389/fchem.2022.865304

**Published:** 2022-04-26

**Authors:** Ming Xu, Li-Le Wang, Gang Chen, Yin-Yun Chen, Dan Liu, Jiang-Tao Liao

**Affiliations:** ^1^ Institute of Translational Medicine, Hunan Provincial People’s Hospital/The First Affiliated Hospital of Hunan Normal University, Changsha, China; ^2^ Department of Gastroenterology Medicine, Hunan Provincial People’s Hospital/The First Affiliated Hospital of Hunan Normal University, Changsha, China; ^3^ Department of Respiratory Medicine, Hunan Provincial People’s Hospital/The First Affiliated Hospital of Hunan Normal University, Changsha, China

**Keywords:** optical sensing, pollutant adsorption, pollutant removal, picronitric acid, COF

## Abstract

Picric acid (PA) is an important chemical product which has been widely used in dye manufacturing, antiseptics, and pharmaceuticals. Owing to PA’s extreme electron-deficient structure, its natural degradation is hard, leading to accumulation in the environment and finally threatening the ecosystem and human health. In this case, PA detection and removal becomes more and more important, concerning environmental protection and human health. In this study, an ionic covalent organic framework (I-COF) was synthesized and modified with a luminescent Tb(III) emitter (Tb(DPA)_3_^3-^, DPA = pyridine-2,6-dicarboxylic acid), *via* ionic exchange. The resulting composite material (Tb-COF) was fully characterized by geometric analysis, IR, XRD, porosity analysis, SEM/TEM, and elemental analysis. It was found that Tb(DPA)_3_^3-^ was loaded into the hexagonal cage in an I-COF host with an ionic exchange ratio of 41%. The as-synthesized Tb-COF showed weak Tb(III) emission and strong red COF emission, after adding PA, Tb(III) emission was increased whereas COF emission weakened greatly, showing sensing behavior. Linear working curves were observed with good selectivity. The sensing mechanism was revealed as follows. PA molecules replaced the [Tb(PDA)_3_]^3-^ component trapped in Tb-COF, releasing free luminescent [Tb(PDA)_3_]^3-^. After incorporating PA in the hexagonal cage, the COF emission was quenched. This sensing mechanism ensured a good selectivity over competing species, including cations, anions, and nitrocompounds. The adsorption and removal performance of I-COF for PA were investigated as well.

## Introduction

Picric acid (PA) is an important chemical product which has been widely used in dye manufacturing, antiseptics, and pharmaceuticals (Kosal et al., 2002; Dinca et al., 2006; Zhang et al., 2014). Owing to PA’s extreme electron-deficient structure, its natural degradation is hard, leading to accumulation in the environment and finally threatening the ecosystem and human health (Zhou et al., 2012; Zhu et al., 2014). In this case, PA detection and removal becomes more and more important, concerning environmental protection and human health. Although modern analytical techniques such as chromatography and electroanalysis are good at PA identification and quantification, they are ineffective in removing PA (Ai et al., 2009; Zou et al., 2014). Its removal generally is performed *via* adsorption of a porous material or chemical reaction with precipitant. It seems that the recognition and removal of PA may be carried out by a one-step procedure using a proper optical sensing platform, where its sensing probe gives a sensing signal for PA and its supporting host adsorbs PA.

As for the sensing probe, PA requires a stable emitter since its electron-deficient structure tends to quench most emitters. It appears that rare earth-based probes are promising ones for PA detection. These metal-centered emitters have long-lived sharp emission lines. Chemical modifications and reactions exert a slim effect on these emission lines, making them a promising sensing probe for PA detection. As for the supporting host, porous materials with stable structure and high surface area-to-volume ratio are desired (Wang et al., 2011). Ever since their successful synthesis, COF (covalent organic framework) materials have been harvesting much research attention owing to their virtues of structural regularity and uniform micropores (Feng et al., 2012; Slater and Cooper, 2015; Waller et al., 2015). COF materials generally are porous polymers based on versatile organic building blocks with monodispsered micropores, which endows COF materials with multiple potentials and applications in optoelectronics, storage and separation, catalysis, and sensing materials (Colson et al., 2011; Dalapati et al., 2013). By changing the type and structure of organic building blocks, the shape, size, and instinct of micropores can be modified and controlled, which makes COF an attractive host material for further application.

There are, however, two issues when changing these organic building blocks. First, the structural change of resulting COF is sometimes unpredictable when changing organic building blocks, since the basic geometry of the core framework may be changed as well. Second, the organic building blocks of COFs are usually neutral ones, so that the resulting COFs are crystalline polymers with no charge, which limits the further modification and grafting of functional groups. For example, in the field of optical sensing, sensing probe should be immobilized and dispersed in supporting host to guarantee fluent dispersal and diffusion of analyte molecules. Although COF materials satisfy most requirements for an ideal supporting host, such as uniform pore size distribution, enough mechanical strength, stable, and versatile structure, there is an important issue for COFs, which is their neutral structure.

Such neutral structure of COFs greatly limits the immobilization of sensing probes in COFs, where sensing probes consequently shall either be adsorbed in COF pores *via* van der Waals’ force or covalently grafted to the organic backbone of COFs (Spitler et al., 2012). The van der Waals’ force is weak, which leads to desorption of the sensing probe from COFs. The covalent grafting between the sensing probe and organic COF backbone is strong enough, but the strong and direct interaction between the sensing probe and organic COF backbone tends to affect COF’s basic geometry and the sensing performance. In addition, such covalent grafting on COFs basically denies the application of metal-based sensing probes in COFs, such as transition metal complexes and rare-earth complexes, which are two classes of important sensing probes. To solve this problem, MOF (metal–organic framework) materials have been proposed, which have similar structural building blocks with COFs, so that transition metal complexes and rare earth complexes can be immobilized in these porous hosts. For example, Zhang et al. (2016a) reported ratiometric detection for an anthrax biomarker using a bio-metal–organic framework as a supporting matrix. According to Li et al., Ln(BTC) MOFs were reported for the ratiometric optical sensing of dipicolinic acid (Zhang et al., 2016b). In addition, these Ln(BTC) MOFs were further modified with organic dyes to realize naked-eye detection of dipicolinic acid (Zhang et al., 2017). Ma et al. reported that the Ru(II) complex-doped metal–organic frameworks for two-photon absorption and singlet oxygen generation (Zhang et al., 2016c).

**SCHEME 1:**
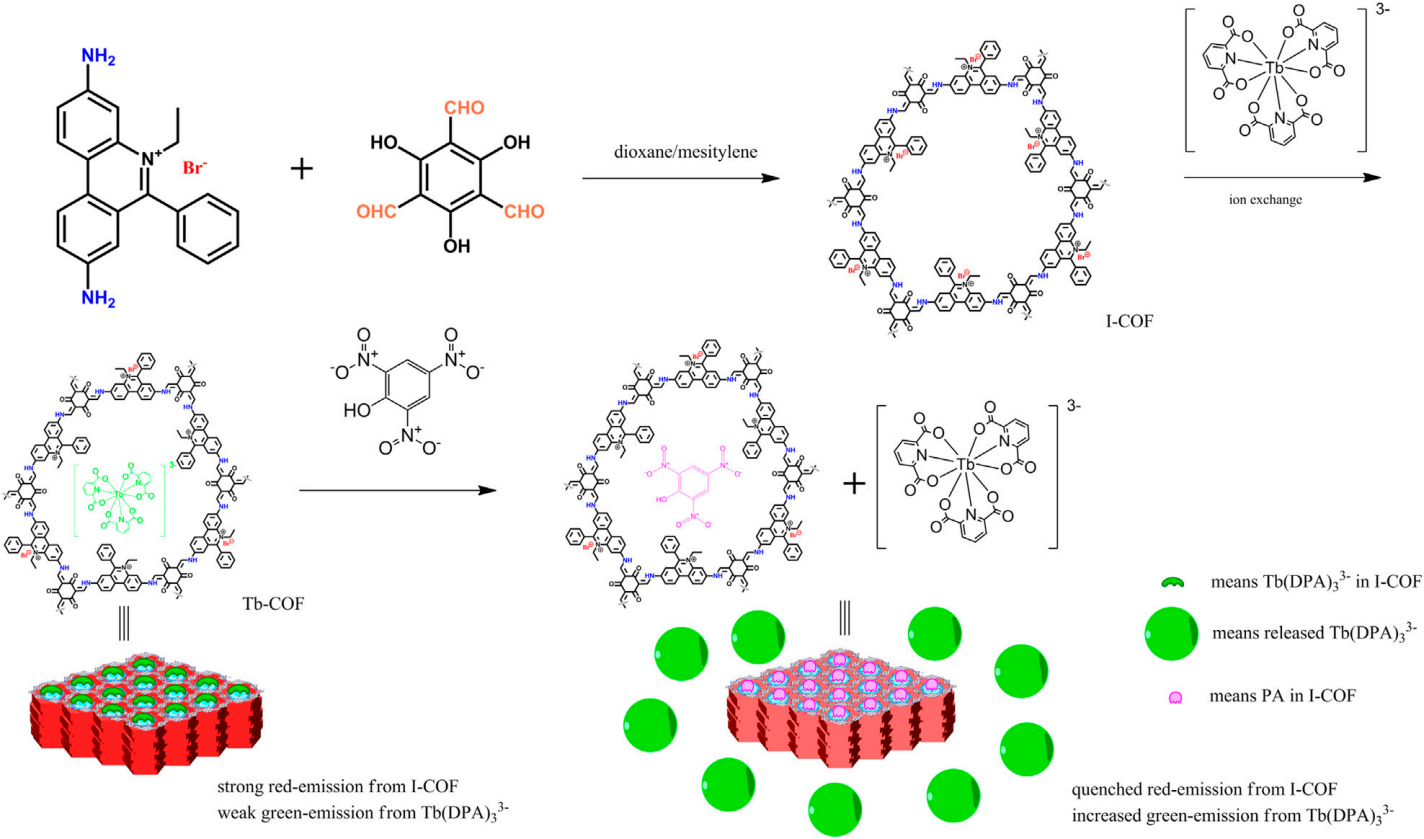
Synthetic route and sensing strategy of I-COF and Tb-COF.

Mandal S. and coworkers have reported and demonstrated a series of well-designed MOF-based materials for the selective sensing/removal of phosphate ions, Hg(II) ions, Fe(III) ions, nitro aromatic explosives, and practical applications (Asha et al., 2014; Asha et al., 2016; Rao and Mandal, 2017; Chandra Rao and Mandal, 2018; Pankajakshan et al., 2018; Pankajakshan et al., 2019; Mandal et al., 2021). These aforementioned results suggest that the microporous crystalline MOF materials are the attractive ones serving as a supporting host for sensing probes. On the other hand, COFs have shown structural diversity as well. As a consequence, new COFs should be developed to conveniently apply transition metal and rare earth complexes as a sensing probe. In this work, an easy protocol was proposed by synthesizing an ionic COF material as host so that the sensing probe could be loaded into this COF host *via* ionic exchange reaction, which was a well-known feature in zeolites (Van Humbeck et al., 2015; Ma et al., 2016). As shown in Scheme 1, the ionic COF, denoted as I-COF, has a cationic backbone, leaving the anionic Br^−^ ready for ionic exchange. As for the sensing probe, Tb(DPA)_3_^3-^ was selected as the sensing probe for ionic exchange. The combination of the ionic COF and Tb(DPA)_3_^3-^ is supposed to realize optical sensing signals for picric acid.

## Experimental Section

### Reagents and Equipment

The synthetic route and sensing strategy of I-COF and Tb-COF is shown in Scheme 1, using 3,8-diamino-5-ethyl-6-phenylphenanthridin-5-ium bromide, 2,4,6-trihydroxybenzene-1,3,5-tricarbaldehyde, and Na_3_Tb(DPA)_3_ as starting compounds. All chemicals used in this work were AR grade ones purchased from Aldrich Chemical Company, including dioxane, mesitylene, 3,8-diamino-5-ethyl-6-phenylphenanthridin-5-ium bromide, picric acid standard sample, pyridine-2,6-dicarboxylic acid (H_2_DPA), NaOH, Tb(Cl_3_)•nH_2_O, phloroglucinol, hexamethylenetetramine, trifluoroacetic acid, and p-toluenesulfonic acid. Organic solvents were purified with standard protocols.

Powder X-ray diffraction (PXRD) was performed on a D/MAX2550 (Riguku) diffractometer using Cu-K radiation at 50 kV and 200 mA. IR spectra were recorded by using a Fourier transform IR spectrometer (IFS 66V/S). Scanning electron microscopy (SEM) and transmission electron microscopy (TEM) images were taken from an S-4800 microscope (Hitachi) and a JEM-2010 microscope (JEOL), respectively. UV–Vis and emission spectra were recorded using a UV-3101 PC spectrometer (Shimadzu) and a F7000 spectrometer (Hitachi), respectively. The N_2_ adsorption and desorption experiment was performed on a Quantachrome Autosorb iQ_2_ analyzer at 77 K (liquid nitrogen). The ^13^C NMR spectrum was recorded using an Avance III 400 WB spectrometer (Bruker, 100.62 MHz, 9.39 T, and 297 K), using adamantine as reference. ^1^H spectra were recorded on this spectrometer with tetramethylsilane as reference (300 MHz). Thermal analysis was performed on a NETZSCH STA 449C thermal analyzer (10°C/min). Elemental analysis was performed by a Carlo Erba 1106 elemental analyzer. XPS (photoelectron spectroscopy) spectra were analyzed on an ESCALAB 250 (Thermo Scientific) spectrometer using Al Ka radiation (1486.6 eV). ICP data were collected by a Shimadzu ICPs-1500D spectrometer. Thermogravimetric analysis (TGA) was performed on a Perkin–Elmer thermal analyzer.

### Synthesis of [Tb(DPA)_3_]^3-^, I-COF, and Tb-COF

The sensing probe Na_3_[Tb(DPA)_3_] was synthesized as follows: TbCl_3_ 6H_2_O (2 mmol) and DPA (6 mmol) were mixed in H_2_O (20 ml). NaOH solution (1 M) was added to adjust the pH to 5.2. After vaporizing solvent water under reduced pressure (100 kPa), the solid product was collected and recrystallized in water. Anal. Calcd. For C_21_H_37_N_3_O_26_Na_3_Tb (Na_3_Tb(PDA)_3_ 14H_2_O): C, 25.86; H, 3.82; and N, 4.31. Found: C, 25.70; H, 3.97; and N, 4.12. Its single crystal structure was obtained and discussed later to confirm its identity.

I-COF was synthesized using 3,8-diamino-5-ethyl-6-phenylphenanthridin-5-ium bromide and 2,4,6-trihydroxybenzene-1,3,5-tricarbaldehyde as starting compounds. 2,4,6-trihydroxybenzene-1,3,5-tricarbaldehyde was first synthesized as follows: a mixture of hexamethylenetetramine (108 mmol), phloroglucinol (48 mmol), and trifluoroacetic acid (90 ml) was stirred under N_2_ stream, then heated at 100°C for 150 h. After cooling, the organic phase was extracted with CH_2_Cl_2_ (100 ml x3) and vaporized to give pale yellow solid. Yield: 11%. Anal. Calcd. For C_9_H_9_O_6_: C, 51.44; H, 2.88; N, 0.0. Found: C, 51.29; H, 2.93; N, 0.05 ^13^C NMR (CDCl_3_) δ 196.2, 172.6, and 103.2.

The above obtained 2,4,6-trihydroxybenzene-1,3,5-tricarbaldehyde (2 mmol) was mixed with 3,8-diamino-5-ethyl-6-phenylphenanthridin-5-ium bromide (3 mmol), and then dissolved in a mixed solvent of dioxane (10 ml), mesitylene (10 ml) and aqueous acetic acid (2 ml, 6 M). This solution was degased and sealed in a Pyrex tube. After being heated at 120°C for 3 days, red-purple solid product was collected, dispersed in THF/ethanol, and stirred for 24 h. The final product was dried at 100^°^C for 12 h to get I-COF. Yield: 72%. Anal. Calcd. For C_162_H_120_N_18_O_12_Br_6_: C, 65.07; H, 4.04; and N, 8.43. Found: C, 65.25; H, 4.17; and N, 8.49. ^13^C NMR (solid) δ 190.5, 167.1, 150.3, 144.7, 135.9, 130.4, 127.8, 113.6, 47.3, and 18.4.

Tb-COF was synthesized *via* anionic exchange with Na_3_[Tb(DPA)_3_] as follows: I-COF (1.5 g) was dispersed in 20 ml of ethanol/H_2_O (v:v = 1:1), then excess Na_3_[Tb(DPA)_3_] (3.0 g) was added. This mixture was stirred at room temperature for 24 h and then filtered off. Such anionic exchange procedure was repeated for another four times. The final product was flushed with water and dried in vacuum at 120°C for 2 days to give Tb-COF. Yield: 85%. Anal. Calcd. For C_162_H_120_N_18_O_12_Br_3_ C_21_H_9_N_3_O_12_Tb_1_ (assuming that 50% of Br^−^ ions were replaced by [Tb(DPA)_3_]^3-^: C, 64.56; H, 3.82; and N, 8.61. Found: C, 64.61; H, 3.90; and N, 8.55. ^13^C NMR (solid) δ 190.6, 169.2, 167.4, 160.0, 150.7, 145.1, 136.4, 134.9, 131.1, 126.5, 114.7, 46.9, and 19.1.

## Methods

For sensing performance evaluation, Tb-COF standard suspension was first prepared by dispersing 0.4 g of Tb-COF in a mixed solvent of ethanol:H_2_O (40 ml, 10 mg/ml, v:v = 1:1). The resulting mixture was treated in ultrasonic bath for 5 min. Then, PA standard was finely weighted and mixed with Tb-COF solution. Before sending to the spectral experiment, each sample was treated in ultrasonic bath for 3 min. For the PA adsorption experiment, Tb-COF solid was finely weighted and mixed with PA standard solution (10 ml, 100 μM). The mixture was treated in ultrasonic bath and then centrifuged (5,000 rpm) for 10 minutes before sending to quantification.

## Results and Discussion

### Geometry of the Probe and I-COF: [Tb(DPA)_3_]^3-^ Single Crystal, I-COF-Simulated Structure, and PXRD

**FIGURE 1:**
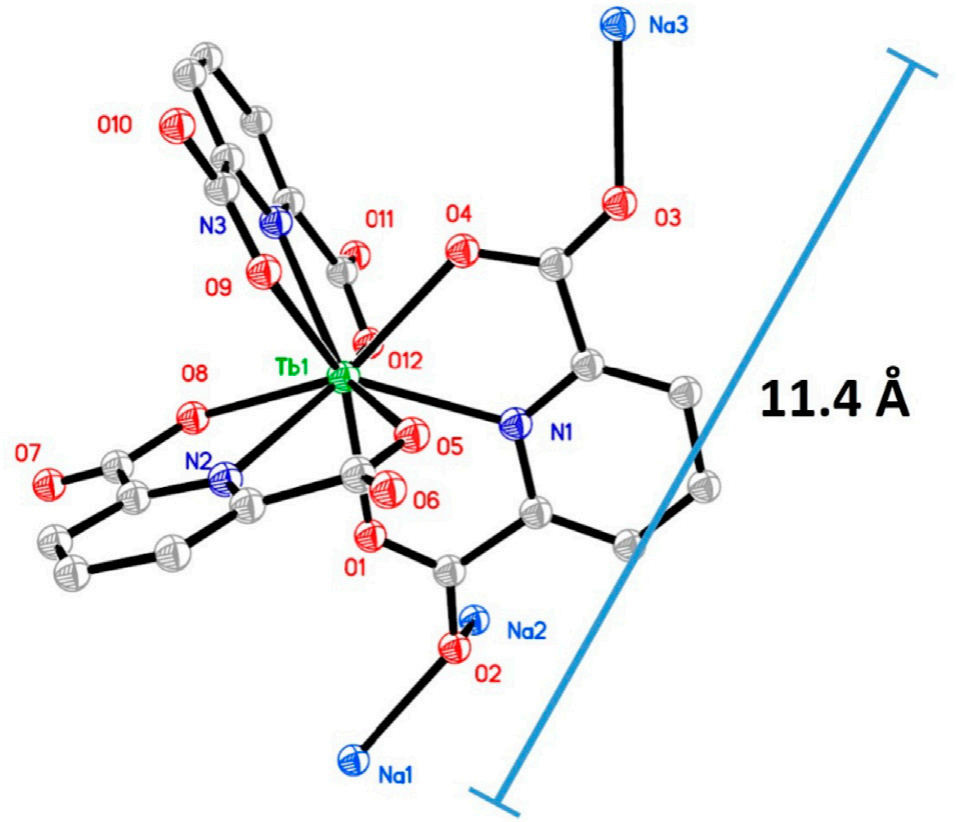
Single crystal structure of Na_3_Tb(PDA)_3_, H atoms and water molecules are omitted for a clear view.

The Na_3_Tb(PDA)_3_ single crystal structure was obtained (CCDC 1185465). Some of its key structural parameters are listed in Table 1. A tetragonal system is adopted by a Na_3_Tb(PDA)_3_ crystal, with slight distortion. There are four [Tb(PDA)_3_]^3-^ components in a cell unit, so that the cell length values (b and c) are as large as ∼18 Å, but the cell length of a is only 9.6 Å. As shown in Figure 1, three tridentate DPA ligands coordinate with a Tb(III) ion, forming a non-coordination field around Tb(III) central ion. The typical coordination bond length and bond angle values in Table 1 are comparable to the literature values of similar single crystals (Li et al., 1993). The counterions (three Na^+^ ions) are randomly distributed outside of the [Tb(PDA)_3_]^3-^ coordination sphere to meet the charge balance. The maximum diameter of [Tb(PDA)_3_]^3-^ is measured as ∼11.4 Å using a Na_3_Tb(PDA)_3_ crystal. The small size of [Tb(PDA)_3_]^3-^ component makes it convenient to be loaded in the COF matrix, which will be confirmed later.

**TABLE 1:** Key structural parameters of an Na_3_Tb(PDA)_3_ single crystal.

Bond length	(Å)	Bond angle	(^o^)	Cell length	(Å)	Cell angle	(^o^)
Tb-O1	2.406	O1-Tb-N1	64.16	a	9.630	α	90.0
Tb-N1	2.504	N1-Tb-O4	64.07	b	18.840	Β	90.5
Tb-O4	2.415	O5-Tb-N2	64.33	c	18.278	γ	90.0
Tb-O5	2.429	N2-Tb-O8	63.86				
Tb-N2	2.509	O9-Tb-N3	64.03				
Tb-O8	2.424	N3-Tb-O12	64.77				
Tb-O9	2.439						
Tb-N3	2.505						
Tb-O12	2.410						

**FIGURE 2:**
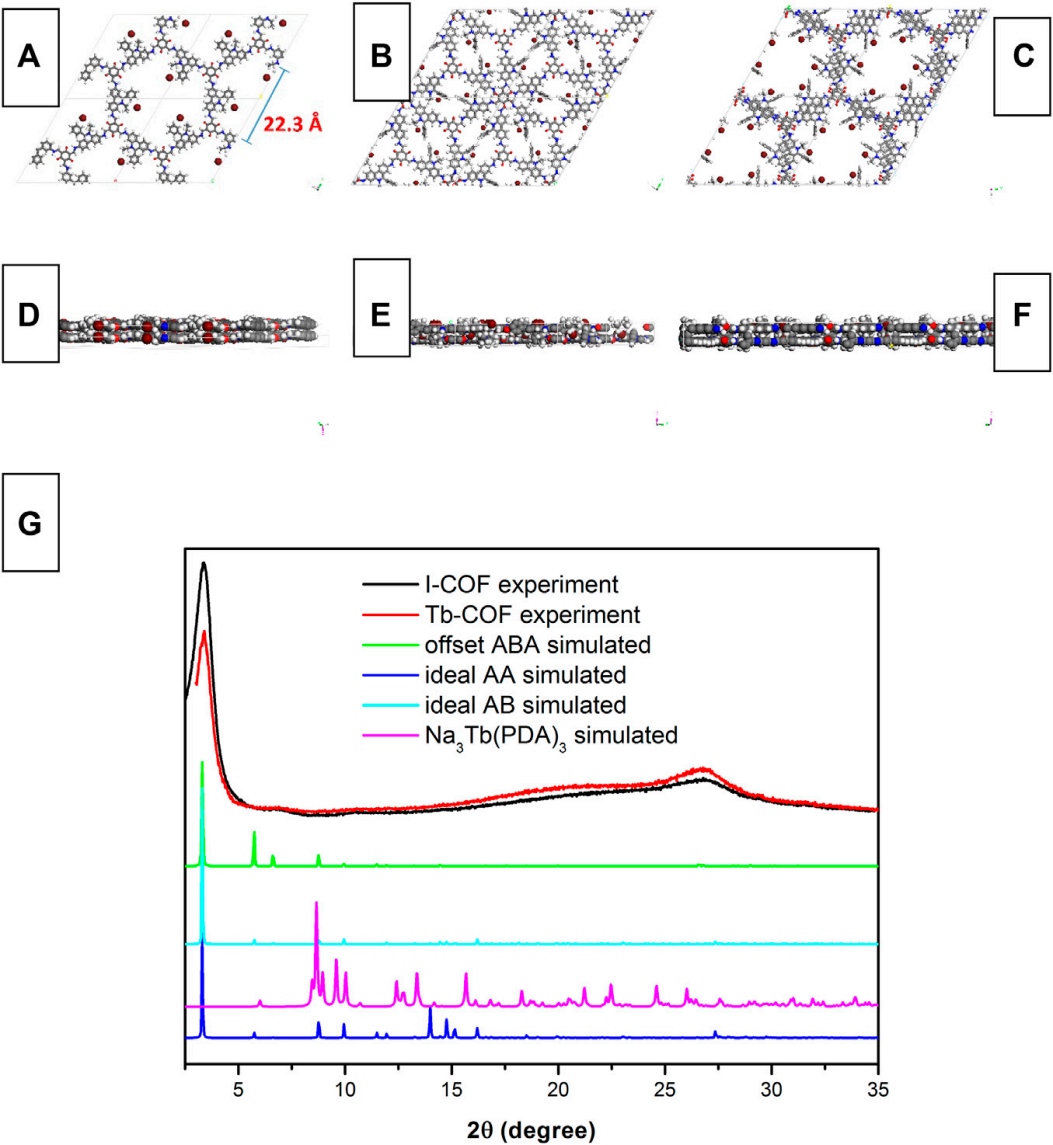
Simulated monolayer structure of I-COF optimized by the universal force field model and its corresponding alignment, **(A)** top view of ideal AA stacking, **(B)** top view of ideal AB stacking, **(C)** top view of offset ABA stacking, **(D)** side view of ideal AA stacking, **(E)** side view of ideal AB stacking, **(F)** side view of offset ABA stacking, **(G)** XRD patterns of I-COF, Tb-COF, ideal AA stacking, ideal AB stacking, offset ABA stacking, and Na_3_Tb(PDA)_3_.

Figure 2 shows the simulated monolayer structure of I-COF optimized by the universal force field model and its corresponding alignment. It is observed from Figure 2A that the diameter of the I-COF monolayer hexagonal cage is 22.3 Å, which is big enough for the [Tb(PDA)_3_]^3-^ component. There are three Br^−^ anions in each hexagonal cage, which allows the ionic exchange of one [Tb(PDA)_3_]^3-^ component to meet the charge balance after ionic exchange. There are three possible alignment modes for I-COF layers, which are ideal AA stacking, ideal staggered AB stacking, and offset ABA stacking. The geometrical energy minimization by the universal force field Forcite module suggests that the offset ABA stacking has the lowest total energy (285 kcal/mol) compared to those of ideal AA stacking (532 kcal/mol) and ideal staggered AB stacking (451 kcal/mol). In this case, it is assumed that the offset ABA-stacking mode is applied by I-COF solid which is energetically favored.

This statement is strengthened by the XRD comparison between I-COF solid and the aforementioned three stacking modes. It is observed from Figure 2 that the PXRD of I-COF solid is similar to the simulated XRD of offset ABA-stacking mode, indicating that I-COF solid tends to take an energetically favored alignment. A sharp diffraction peak of 3.3° and a broad one of 27° are observed, corresponding to the (100) and (001) reflections. Such ABA-stacking mode, however, may compromise the loading capacity since each I-COF monolayer is covered up by the neighboring upper and lower I-COF layers, which will be discussed later. As for Tb-COF, its PXRD is nearly identical compared to I-COF, with no peaks from dopant Na_3_Tb(PDA)_3_, indicating that the I-COF matrix has been well preserved after ionic exchange.

### Composition of Tb-COF: IR, N_2_ Adsorption/Desorption, SEM, TEM, and EDX

**FIGURE 3:**
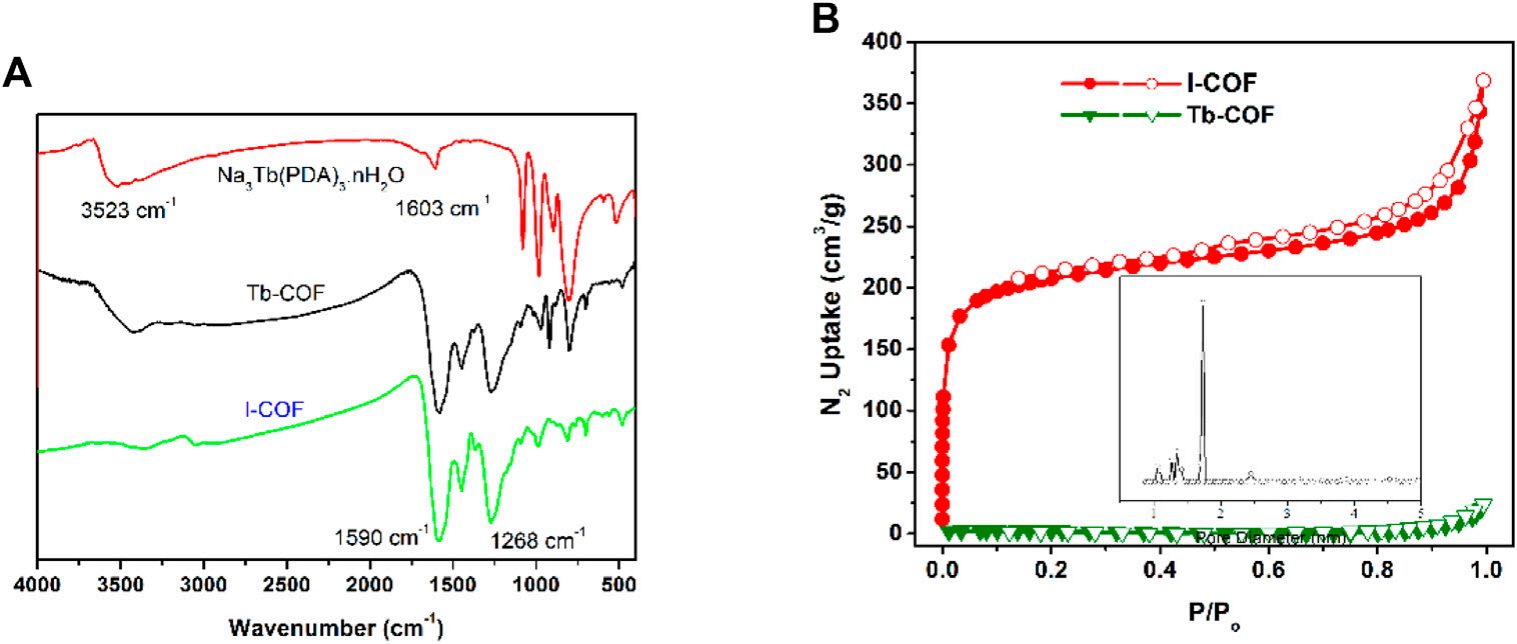
**(A)**: IR spectra of I-COF, Tb-COF, and Na_3_Tb(PDA)_3_. **(B)**: N_2_ adsorption and desorption isotherms of I-COF and Tb-COF. Inset: pore size distribution of I-COF.

The IR spectra of I-COF, Tb-COF, and Na_3_Tb(PDA)_3_ are shown in Figure 3. As for Na_3_Tb(PDA)_3_, the broad IR bands around 3,523 cm^−1^ and 1,603 cm^−1^ are attributed to C=O vibration and stretching. As for I-COF, the strong IR bands at 1,590 cm^−1^ and 1,268 cm^−1^ are named as vibrations from C=C and C-N bonds. These characteristic bands are all found from the IR spectrum of Tb-COF. There are no new IR bands, indicating that Na_3_Tb(PDA)_3_ has been well-preserved in Tb-COF.

The successful loading of Na_3_Tb(PDA)_3_ in I-COF is first confirmed by porosity comparison between I-COF and Tb-COF. As shown in Figure 3, a sharp N_2_ uptake is observed for I-COF at a low pressure of 0–0.1 P/P_0_, which reveals its microporous network. The Brunauer–Emmett–Teller (BET) surface area of I-COF is measured as 770 m^2^/g, with a pore size of ∼17.0 Å. This value is smaller than the diameter of the simulated I-COF monolayer hexagonal cage (22.3 Å), but still larger than the maximum diameter of [Tb(PDA)_3_]^3-^ (∼11.4 Å). In this case, it is assumed that, theoretically, there will be only one [Tb(PDA)_3_]^3-^ component in each I-COF hexagonal cage, to meet room requirement and charge balance. After loading procedure, [Tb(PDA)_3_]^3-^ component occupies I-COF hexagonal cages, resulting in the rather small BET surface of Tb-COF (<10 m^2^/g).

**FIGURE 4:**
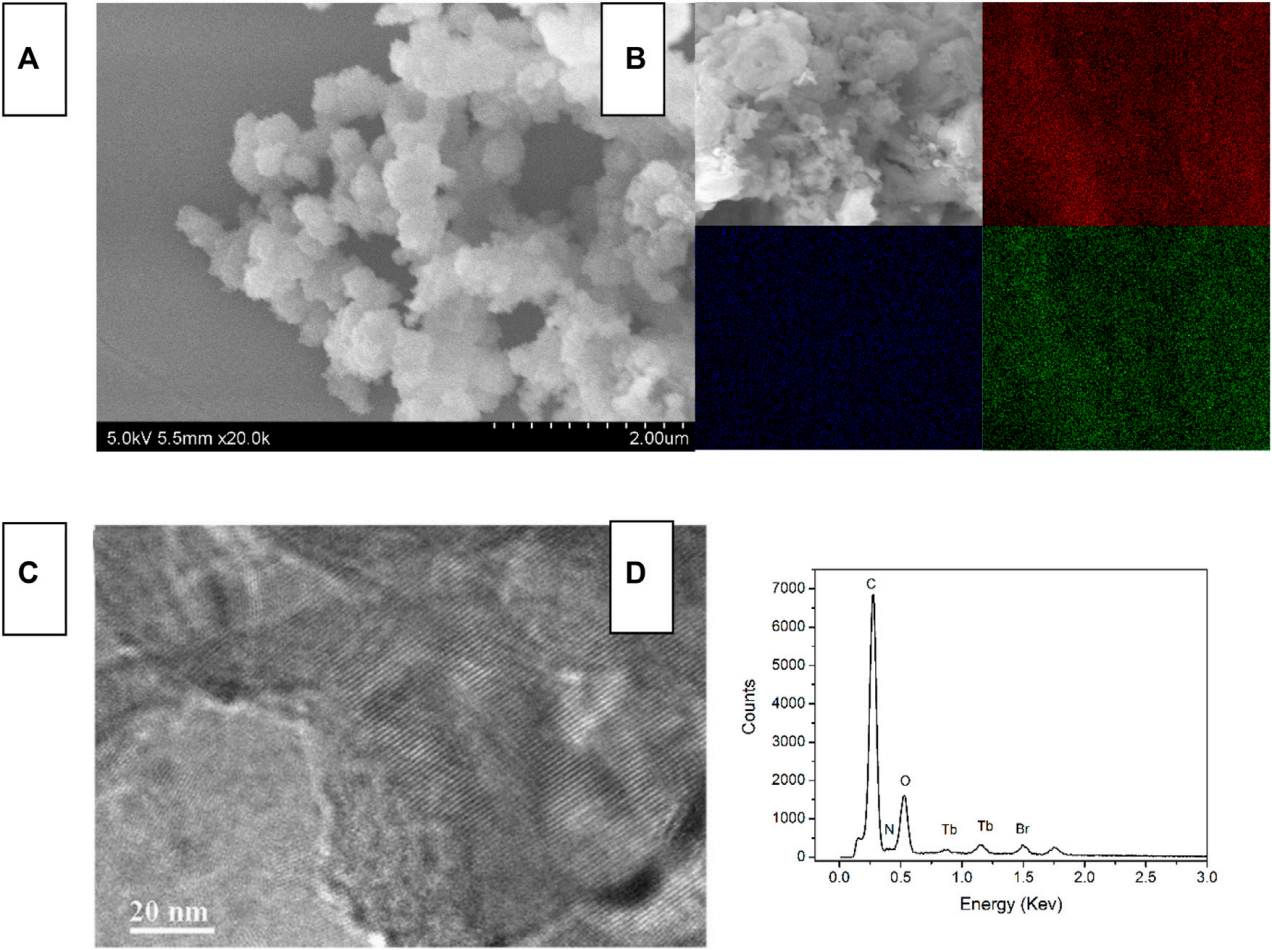
SEM image **(A)**, elemental mapping [**(B)**, red = C, blue = N, and green = Tb], TEM **(C)** and EDX **(D)** of Tb-COF.

The SEM/TEM images of Tb-COF, along with its elemental mapping and EDX data, are shown in Figure 4. Tb-COF shows a spherical morphology, with diameter ranging from 20 to ∼50 nm. The highly ordered hexagonal structure is clearly observed. Its EDX data suggest that there are five elements in Tb-COF, C, N, O Tb, and Br, which are consistent with the elemental composition of Tb-COF. Its elemental mapping shown in Figure 2 indicates that Tb element is uniformly distributed in the whole Tb-COF sample, instead of being concentrated on the surface, which means that the [Tb(PDA)_3_]^3-^ component has been incorporated into the hexagonal cage of I-COF, forming Tb-COF. No obvious signal from Na indicates the successful ionic exchange. On the other hand, Br element is still observed in Tb-COF, indicating an incomplete ionic exchange between the Br^−^ and [Tb(PDA)_3_]^3-^ components.

The elemental analysis data of Tb-COF are measured as C, 64.61; H, 3.90; and N, 8.55. Upon a 100% ionic exchange, the molecular composition should be C_162_H_120_N_18_O_12_∙2[C_21_H_9_N_3_O_12_Tb_1_], with an elemental analysis of C, 64.15; H, 3.64; and N, 8.80. As aforementioned, I-COF allows only one [Tb(PDA)_3_]^3-^ component in each hexagonal cage, which is 50% ionic exchange, and its molecular composition should be C_162_H_120_N_18_O_12_Br_3_∙C_21_H_9_N_3_O_12_Tb_1_, with an elemental analysis of C, 64.56; H, 3.82; and N, 8.61. Clearly, the experimental data support a 50% of ionic exchange. For a more direct evaluation on the doping level, the Br and Tb contents of Tb-COF are determined as 9.8 and 3.2 ppm, respectively, corresponding to a molar ratio of 6.08:1 and suggesting an ionic exchange ratio of 33% (vs. total Br^−^). This low doping level is explained by the ABA-stacking mode of I-COF which compromises the loading capacity since each I-COF monolayer is covered up by the neighboring upper and lower I-COF layers, as aforementioned.

**FIGURE 5:**
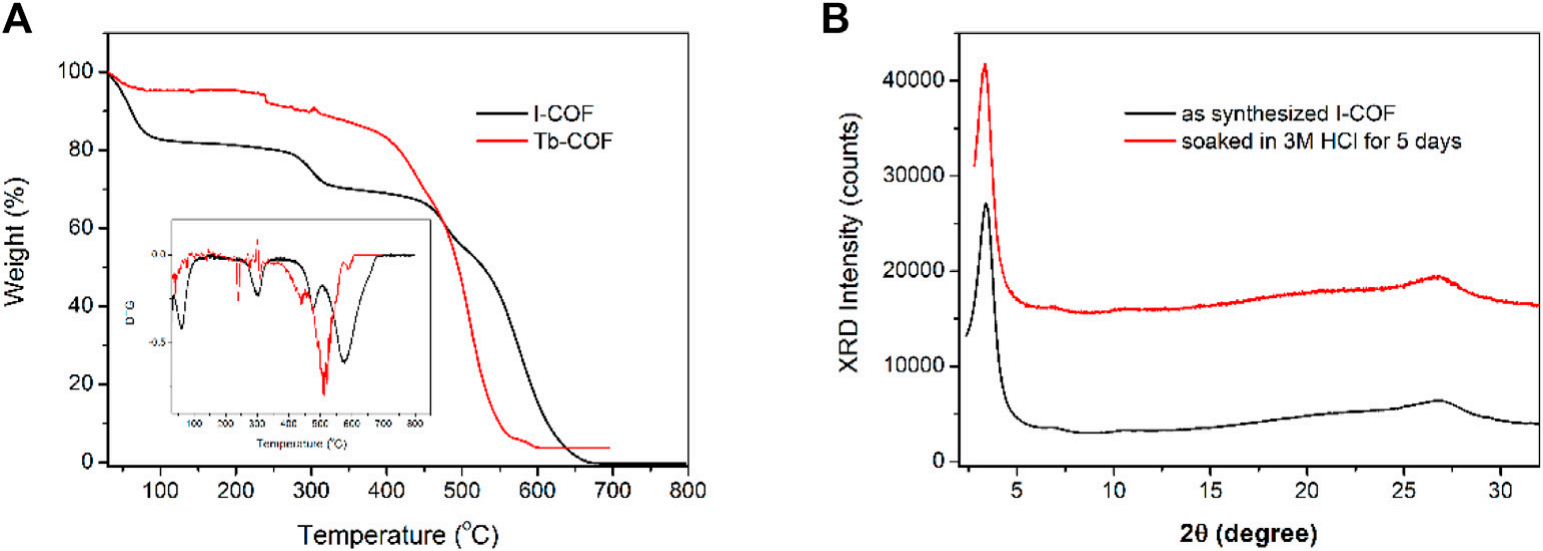
**(A)**: TGA and DTG of I-COF and Tb-COF. **(B)**: XRD patterns of I-COF before and after being soaked in 3M HCl for 5 days.

The thermal stability of I-COF and Tb-COF is investigated *via* their thermogravimetric analysis (TGA) curves shown in Figure 5. As for I-COF, there are four endothermic peaks at 62°C, 302°C, 477°C, and 575°C, respectively. I-COF is fully decomposed at 600°C, with no residual. The first one is attributed to the thermal evaporation of adsorbed H_2_O, and the following three are assigned as thermal degradation of the I-COF framework. As for Tb-COF, there is no obvious weight loss of adsorbed H_2_O. This is because the ionic exchange procedure removes most adsorbed H_2_O molecules. There are three endothermic peaks at 240°C, 440°C, and 511°C, respectively, corresponding to the thermal degradation in the Tb-COF framework. At 600°C, Tb-COF weight residual is measured as 3.7% which is assigned as the metal residual (Tb oxide, 3.3%). The incorporation of [Tb(PDA)_3_]^3-^ component tends to move these endothermic peaks to lower temperature. Nevertheless, these endothermic peaks higher than 200°C indicate that Tb-COF is thermally stable enough for later application. Even being soaked in HCl solution (3 M), this hexagonal structure is still well-preserved, as shown in Figure 5. This good stability endows Tb-COF with a potential in the optical sensing for PA.

### PL Emission Response of Tb-COF Toward PA

**FIGURE 6:**
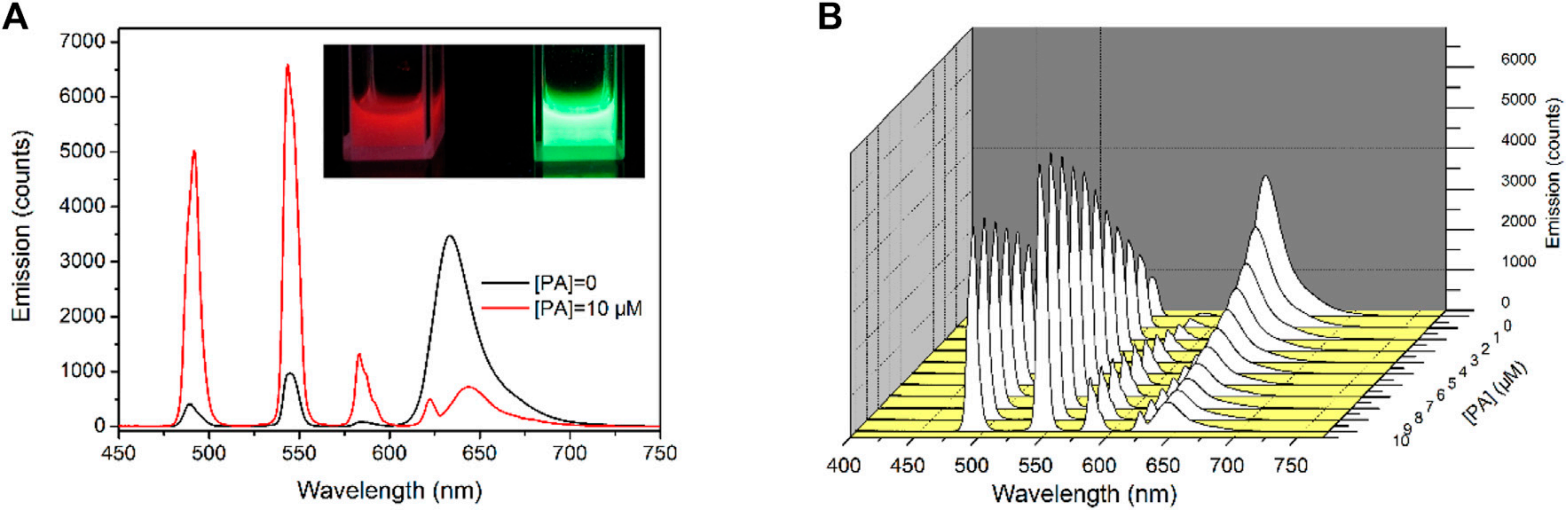
**(A)**: Emission spectra of Tb-COF with PA concentration of 0 and 10 μM, inset: corresponding photo, **(B)**: emission spectra of Tb-COF with increasing PA concentrations from 0 to 10 μM.

It has been previously confirmed that Tb-COF is stable enough in acidic medium and thus can be developed for PA optical sensing. To assess the sensing performance of Tb-COF, its emission spectra upon increasing PA concentrations are considered. As shown in Figure 6, in the absence of PA, Tb-COF (dispersed in ethanol, 5 mg/ml) shows a strong red emission peaking at 634 nm and four weak emission lines from Tb(III) peaking at 492, 543, 583, and 623 nm. The red emission is attributed to the π–π* excited state of the organic framework in Tb-COF, which is consistent with its long conjugation chain. The antenna energy transfer from DPA ligand to Tb(III) in Tb(DPA)_3_^3-^ is partially compromised since the DPA-excited state tends to transfer its energy to a surrounding lower energy level of the COF π–π* excited state, leading to the strong π–π* red emission and weak Tb(III) emission as aforementioned (Zhang et al., 2010). Given PA concentration of 10 μM, the four Tb(III) emission lines are obviously increased, while the π–π* red emission is decreased effectively. No spectral shift is detected for the four Tb(III) emission lines owing to their metal-centered *f–f* transitions. The π–π* red emission, however, is not only weakened but also red shifted by ∼12 nm (645 nm) upon the PA concentration of 10 μM, which shall be attributed to the strong electron-withdrawing effect of PA. The presence of PA favors Tb(III) emission and compromises π–π* red emission, showing ratiometric signals and favoring sensing application.

### Working Curves of Tb-COF

For a better understanding on Tb-COF sensing behavior toward PA, its emission intensity variation on increasing PA concentrations are analyzed as follows. According to Valeur et al., if the donor and the acceptor follow a stoichiometry ratio of 1:1, the absorbance or fluorescence intensity shall follow an equation of Eq. 1, where X means absorbance or fluorescence intensity, X_lim_ is the critical value of 100% complexation, and X_0_ is the initial value (Bourson et al., 1993).

$$\text{X}=\text{X0}+\frac{X_{\lim}-X_{0}}{2C_{0}}\times \left\{C_{0}+C_{M}+\frac{1}{Ks}-{\left[{\left(C_{0}+C_{M}+\frac{1}{Ks}\right)}^{2}-4C_{0}C_{M}\right]}^{1/2}\right\}$$ (1)

**FIGURE 7:**
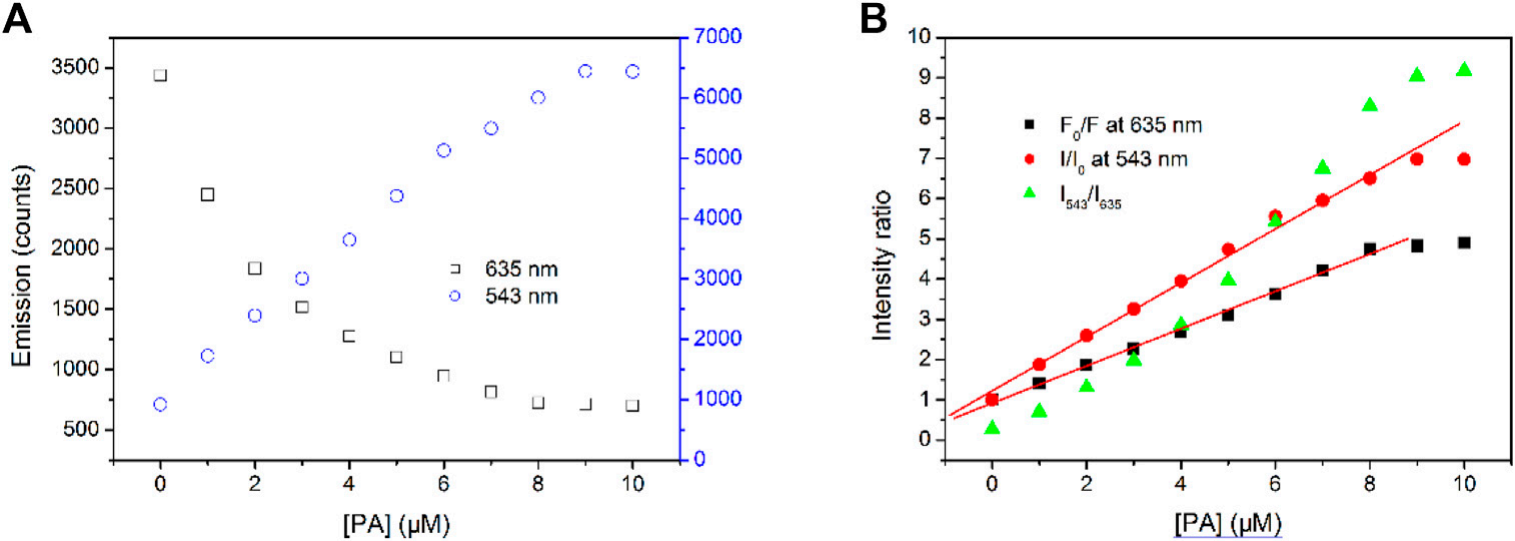
**(A)**Emission intensity variation at 543 and 635 nm of Tb-COF with increasing PA concentrations from 0 to 10 μM, **(B)** intensity ratios of F_0_/F, I/I_0_, and I_543_/I_635_.

An ideal curve of Eq. 1 should be a down-bending curve with increasing acceptor concentrations. This, however, is not the case for the emission intensity variation at 543 nm, which is more like a linear one, as shown in Figure 7. It has been previously confirmed that the hexagonal cage of I-COF allows only one [Tb(PDA)_3_]^3-^ component. Considering the similar molecular sizes of [Tb(PDA)_3_]^3-^ and PA, it is assumed that each hexagonal cage of I-COF fits only one PA molecule as well, which is a stoichiometry ratio of 1:1 as well. In this case, the emission intensity at 543 nm is analyzed *via* Stern–Volmer equation, described by Eq. 2, where I is the emission intensity at 543 nm, I_0_ is the emission intensity without any quencher (PA), and K_s_ is the complexation constant

$${\text{I/I}}_{0}=\text{1}+\text{Ks}\left[\text{PA}\right]$$ (2)

The fitting equation is obtained as I/I_0_ = 1.223 + 6.709 × 10^5^ M^−1^(PA), R^2^ = 0.996, within (PA) the region of 0–9 μM. The limit of detection (LOD, defined as 3σ/N) value is calculated as 0.8 μM (15–17). Upon a higher [PA] value, a down-bending tendency is observed, indicating a 100% complexation between Tb-COF and PA.

On the other hand, the emission intensity at 635 nm follows a non-linear decreasing curve. The residual emission intensity against quencher (PA) concentration shall be explored by another form of Stern–Volmer equation described as Eq. 3. Here, F_0_ means the initial Tb-COF emission intensity at 635 nm without PA, F is the emission intensity at 635 nm, and K_s_^′^ is the complexation constant.

$${\text{F}}_{\text{0}}\text{/F}=\text{1}+\text{Ks′}\left[\text{PA}\right]$$ (3)

The fitting equation is obtained as F_0_/F = 0.917 + 4.637×10^5^ M^−1^(PA), R^2^ = 0.998, within the (PA) region of 0–8 μM. Upon a higher (PA) value, a down-bending tendency is observed, indicating a 100% complexation between Tb-COF and PA.

The ratiometric value I_543_/I_635_ shows a down-bending curve with increasing (PA), instead of linear ones reported by literatures (15–17). This result indicates that the sensing mechanism of Tb-COF toward is not the energy competing between Tb-COF emissive center (635 nm) and Tb(III) ion (543 nm), although such energy competing mechanism has been reported and confirmed.

### Sensing Mechanism of Tb-COF

The aforementioned analysis on working curves of Tb-COF has denied the sensing mechanism of energy competing between Tb-COF emissive center (635 nm) and Tb(III) ion (543 nm). Based on the below two facts, 1) I/I_0_ of Tb(III) emission follows linear increasing correlation with (PA) and 2) F_0_/F of Tb-COF π–π* emission follows linear increasing correlation with (PA), it is assumed that the amount of Tb(III) emitter is linearly increased by (PA), while Tb-COF π–π* emission is just quenched by PA. A possible sensing mechanism is thus proposed and shown in Scheme 1. Here, PA molecules replace the [Tb(PDA)_3_]^3-^ component trapped in Tb-COF, releasing free luminescent [Tb(PDA)_3_]^3-^. After incorporating PA in the hexagonal l cage, the π–π* emission is quenched, leading to decreased emission intensity at 635 nm. The emission decay dynamics of Tb-COF at 543 and 635 nm were recorded. Single-exponential decay patterns were observed as shown in Supplementary Figure S1, corresponding lifetime values were determined as 246.0 µs (at 543 nm, (PA) = 0 μM), 382.7 µs [at 543 nm, (PA) = 5 μM], 488.6 µs [at 543 nm, (PA) = 10 μM], 9.22 ns [at 635 nm, (PA) = 0 μM], 7.38 ns [at 635 nm, (PA) = 5 μM], and 6.19 ns [at 635 nm, (PA) = 10 μM], respectively. It is observed that Tb excited state lifetime is increased with increasing (PA), while that of Tb-COF π–π* emission is decreased with increasing (PA). Considering that both emission components are affected by varying (PA), it is concluded that a dynamic behavior is responsible for the sensing experiment. This dynamic behavior is further confirmed by the absorption spectra of Tb-COF with and without PA. As shown by Supplementary Figure S2, after adding PA into Tb-COF, the absorption spectrum of Tb-COF (with PA) is rather similar compared to Tb-COF without PA, except for the additional PA absorption. There is no new absorption band, suggesting that no new compound or species is yielded after adding PA. This observation further confirms the aforementioned dynamic mechanism.

**FIGURE 8:**
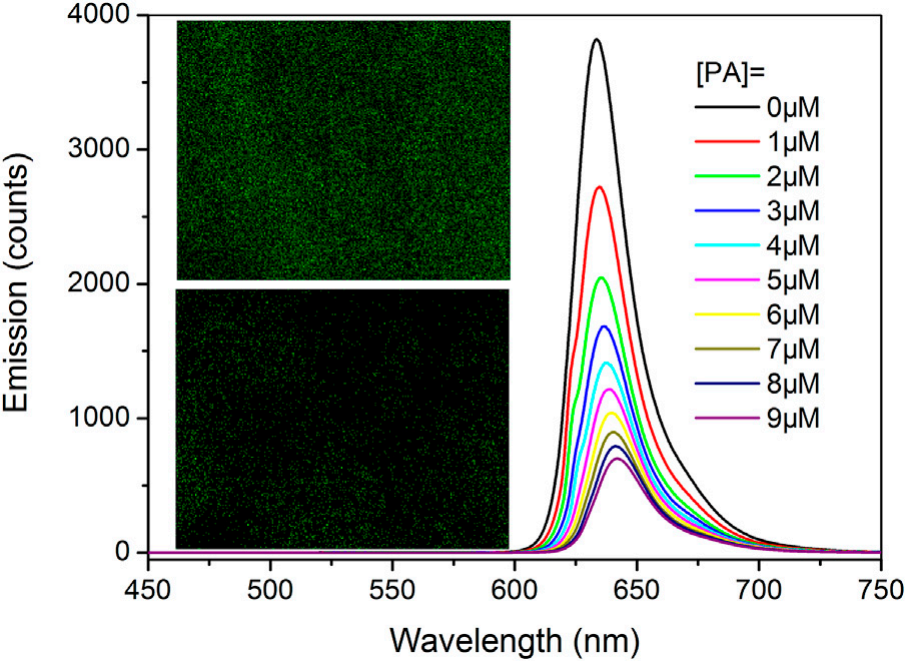
I-COF emission intensity against increasing (PA). Inset: photos of Tb-COF before and after being treated with PA (10 μM).

For a tentative evaluation on this sensing mechanism, I-COF emission intensity against increasing (PA) is shown in Figure 8. Here, similar emission quenching is observed, along with the emission red shift. A Tb elemental mapping of Tb-COF after being treated with PA (10 μM) is shown in Figure 8. Clearly, the Tb content is much less than that in original Tb-COF, indicating that the [Tb(PDA)_3_]^3-^ component has been released upon the presence of PA. In this case, the sensing mechanism is confirmed.

### Selectivity of Tb-COF

**FIGURE 9:**
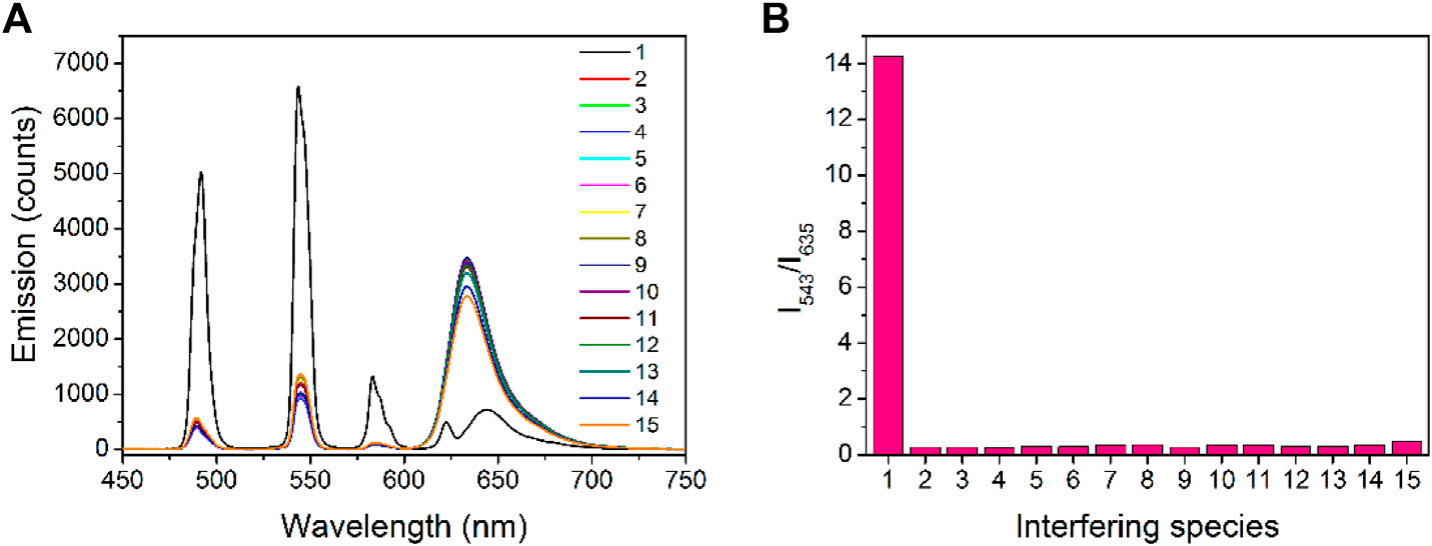
**(A)** Emission spectra of Tb-COF in the presence of PA and interfering species (10 μM), 1, PA; 2, K^+^; 3, Ca^2+^; 4, Mg^2+^; 5, Fe^2+^; 6, Hg^2+^; 7, CO_3_^2-^; 8, ACO^−^; 9, Cl^−^; 10, SO_4_^−^; 11, NO_3_^−^; 12, *p*-nitrobenzene; 13, 2,4-dinitrotoluene; 14, 2,4,6-trinitrotoluene; and 15, phosphate anion. Right: corresponding emission intensity ratios of I_543_/I_635_.

The selectivity performance of Tb-COF toward PA is explored by comparing its emission spectrum with those under interfering ions and compounds, including K^+^, Ca^2+^, Mg^2+^, Fe^2+^, Hg^2+^, CO_3_^2-^, ACO^−^, Cl^−^, SO_4_^−^, NO_3_^−^, *p*-nitrobenzene, 2,4-dinitrotoluene, and 2,4,6-trinitrotoluene. It is observed from Figure 9 that only the presence of PA makes Tb(III) emission (543 nm) increase, with π–π* emission (635 nm) quenched. All aforementioned ions and compounds fail to trigger such phenomenon, which can be explained by the sensing mechanism of Tb-COF. As for cationic ions, they are unable to ionic exchange with [Tb(PDA)_3_]^3-^ component, so that no [Tb(PDA)_3_]^3-^ emitter is released, showing stable Tb(III) emission (543 nm). Although some cationic ions such as Fe^2+^ and Hg^2+^ can quench π–π* emission (635 nm), the quenching effect is still minor. As for anionic ions, they may partially release the [Tb(PDA)_3_]^3-^ emitter, leading to improved Tb(III) emission (543 nm), but π–π* emission (635 nm) is not effectively quenched. In addition, owing to the small size of these interfering anionic ions, the [Tb(PDA)_3_]^3-^ emitter shall not be fully released and may co-exist in the COF hexagonal cage. As for the nitrobenzene compounds, they are neutral ones and impossible to be exchanged with [Tb(PDA)_3_]^3-^, even though they are also electron-deficient ones and quench π–π* emission (635 nm). Since no [Tb(PDA)_3_]^3-^ emitter is released, no Tb(III) emission is increased. The good selectivity of Tb-COF toward PA over these interfering ions and compounds is clearer when comparing their ratiometric values (I_543_/I_635_), as shown in Figure 9. The good selectivity benefits from the sensing mechanism which requires both the replacing/releasing [Tb(PDA)_3_]^3-^ emitter and quenching π–π* emission (635 nm).

### Adsorption and Removal Feature of I-COF

After checking the sensing mechanism of I-COF toward PA, it is found that PA molecules are captured and immobilized in the COF hexagonal cage, which endows I-COF with adsorption and removal feature for PA as well. Aiming at an evaluation on the adsorption performance of I-COF, 100 mg of I-COF wad dispersed in ethanol (5 ml), then PA was added for adsorption. Later, I-COF was centrifuged, solution was extracted, and PA residual was determined by chromatography. The retrieved I-COF was sent to repeat aforementioned procedure. The total adsorbed PA amounts are listed in Table 2. The maximum adsorption of I-COF for PA is ∼7.4 mg/100 mg. This value is close to the theoretical capacity of the I-COF hexagonal cage (7.7 wt%). Hereby, the adsorption feature of I-COF for PA is thus confirmed.

**TABLE 2:** Adsorption performance of I-COF toward PA.

PA added (mg)	PA Adsorbed			
	First cycle	second cycle	Third cycle	Total
5	3.8	0.9	0.1	4.8
10	6.0	1.3	0.1	7.4
20	6.1	1.2	0.1	7.3
30	6.2	1.2	0.1	7.5

100 mg of I-COF, wad dispersed in ethanol (5 ml), then PA was added for adsorption. Later, I-COF was centrifuged, solution was extracted, and PA residual was determined by chromgraphy. The retrieved I-COF was sent to repeat the aforementioned procedure.

## Conclusion

In this work, an ionic COF (I-COF) was synthesized, then the Tb(III) emitter Tb(DPA)_3_^3-^ was loaded onto the I-COF hexagonal cage *via* ionic exchange procedure. The resulting composite material (Tb-COF) was fully characterized by geometric analysis, IR, XRD, porosity analysis, SEM/TEM, and elemental analysis. It was found that Tb(DPA)_3_^3-^ was loaded into the hexagonal cage in the I-COF host with ionic exchange ratio of 41%. Tb-COF was stable enough for PA sensing as suggested by TGA and the acidic soaking experiment. The as-synthesized Tb-COF showed weak Tb(III) emission and strong red COF emission, after adding PA, Tb(III) emission was increased whereas COF emission weakened greatly, showing sensing behavior. Linear working curves were observed with good selectivity. PA molecules replaced the [Tb(PDA)_3_]^3-^ component trapped in Tb-COF, releasing free luminescent [Tb(PDA)_3_]^3-^. After incorporating PA in the hexagonal cage, the COF emission was quenched. This sensing mechanism ensured a good selectivity over competing species, including cations, anions, and nitrocompounds. The adsorption and removal performance of I-COF for PA was investigated as well. For the latter experiment, the dopant adsorption capacity in COF should be improved.

## Data Availability

The datasets presented in this study can be found in online repositories. The names of the repository/repositories and accession number(s) can be found in the article/Supplementary Material.
