# Supplementary material for: A Red-Emitting COF Ionic Exchanged With Green-Emitting Tb(III) Complex Anion: Synthesis, Characterization, Ratiometric Emission Sensing, and Removal of Picric Acid
Source: Front Chem. 2022 Apr 26;10:865304. doi: 10.3389/fchem.2022.865304 (PMC9086536; doi:10.3389/fchem.2022.865304)

Supplementary Material

# Detailed geometric parameters

data_123

_symmetry_cell_setting monoclinic

_symmetry_space_group_name_H-M 'P 21/c'

_symmetry_Int_Tables_number 14

loop_

_symmetry_equiv_pos_site_id

_symmetry_equiv_pos_as_xyz

1 x,y,z

2 -x,1/2+y,1/2-z

3 -x,-y,-z

4 x,1/2-y,1/2+z

_cell_length_a 9.630(1)

_cell_length_b 18.840(3)

_cell_length_c 18.278(2)

_cell_angle_alpha 90

_cell_angle_beta 90.57(1)

_cell_angle_gamma 90

_cell_volume 3316

loop_

_atom_site_label

_atom_site_type_symbol

_atom_site_fract_x

_atom_site_fract_y

_atom_site_fract_z

Tb1 Tb 0.11617(3) 0.23968(2) 0.45413(2)

O1 O -0.0805(5) 0.1622(3) 0.4384(3)

O2 O -0.2175(5) 0.0918(3) 0.3770(3)

O3 O 0.4156(5) 0.2423(3) 0.2673(3)

O4 O 0.2919(5) 0.2696(3) 0.3660(3)

O5 O 0.2583(5) 0.1332(3) 0.4569(3)

O6 O 0.3829(5) 0.0551(3) 0.5210(3)

O7 O -0.1351(6) 0.2767(3) 0.6581(3)

O8 O -0.0425(5) 0.2817(3) 0.5467(3)

O9 O 0.3209(5) 0.2681(3) 0.5281(3)

O10 O 0.4672(6) 0.3458(4) 0.5799(3)

O11 O -0.0937(7) 0.4279(3) 0.3492(4)

O12 O -0.0326(5) 0.3189(3) 0.3857(3)

N1 N 0.0979(5) 0.1779(3) 0.3331(3)

N2 N 0.1157(6) 0.1718(3) 0.5722(3)

N3 N 0.1711(6) 0.3690(3) 0.4679(3)

C1 C -0.1106(7) 0.1273(5) 0.3812(4)

C2 C -0.0034(7) 0.1308(4) 0.3210(4)

C3 C -0.0044(8) 0.0873(4) 0.2596(4)

C4 C 0.1035(9) 0.0924(5) 0.2113(4)

C5 C 0.2084(8) 0.1420(4) 0.2231(4)

C6 C 0.2010(7) 0.1839(4) 0.2853(4)

C7 C 0.3121(7) 0.2367(4) 0.3065(4)

C8 C 0.2898(7) 0.1000(4) 0.5152(4)

C9 C 0.2069(7) 0.1187(4) 0.5822(4)

C10 C 0.2278(8) 0.0870(4) 0.6495(4)

C11 C 0.1502(9) 0.1106(5) 0.7082(4)

C12 C 0.0572(8) 0.1660(4) 0.6982(4)

C13 C 0.0422(7) 0.1949(4) 0.6289(4)

C14 C -0.0585(7) 0.2562(4) 0.6150(4)

C15 C 0.3620(6) 0.3301(5) 0.5427(4)

C16 C 0.2777(8) 0.3904(4) 0.5100(4)

C17 C 0.3093(9) 0.4616(5) 0.5187(5)

C18 C 0.2320(10) 0.5108(5) 0.4817(7)

C19 C 0.1240(10) 0.4887(5) 0.4375(6)

C20 C 0.0945(8) 0.4166(4) 0.4324(4)

C21 C -0.0211(8) 0.3863(4) 0.3849(4)

Na1 Na -0.4036(3) 0.0644(2) 0.4531(2)

Na2 Na -0.3990(3) 0.1603(2) 0.3068(2)

Na3 Na 0.5707(4) 0.3389(2) 0.2506(2)

O13 O -0.5436(8) 0.0612(4) 0.3407(4)

O14 O -0.3098(6) 0.3077(3) 0.4876(3)

O15 O -0.3410(6) 0.1238(8) 0.1865(3)

O16 O 0.5116(7) 0.3692(3) 0.3749(4)

O17 O -0.2480(7) 0.1090(4) 0.5490(4)

O18 O -0.4491(5) 0.1878(3) 0.4314(3)

O19 O -0.2460(5) 0.2597(3) 0.2921(3)

O20 O -0.4090(10) -0.0219(5) 0.2099(5)

O21 O 0.5385(8) 0.1857(5) 0.6268(5)

O22 O -0.7293(9) -0.0682(5) 0.2729(6)

Figure S1. Emission decay dynamics at 543 nm (left chart) and 635 nm (right chart). Single exponential decay patterns were observed, corresponding lifetime values were determined as

543 nm: 246.0 μs ([PA]=0 μM), 382.7 μs ([PA]=5 μM), 488.6 μs ([PA]=10 μM)

635 nm: 9.22 ns ([PA]=0 μM), 7.38 ns ([PA]=5 μM), 6.19 ns ([PA]=10 μM)


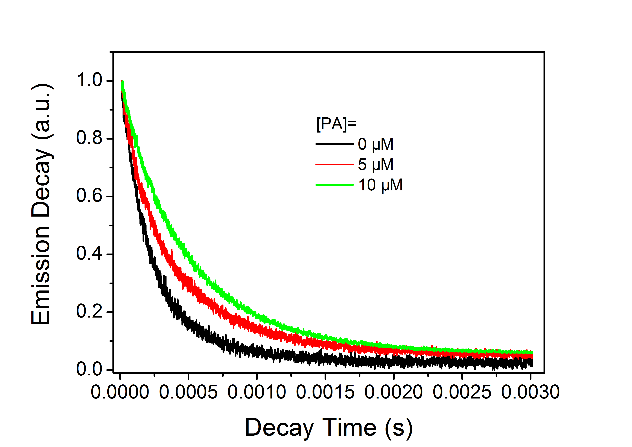

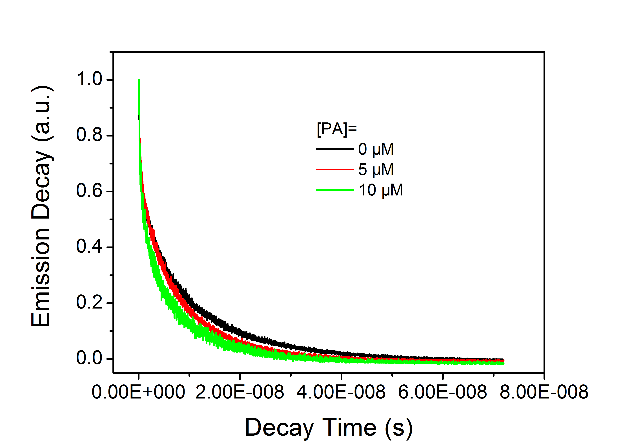


Figure S2. Absorption spectra of PA and Tb-COF with or without PA.


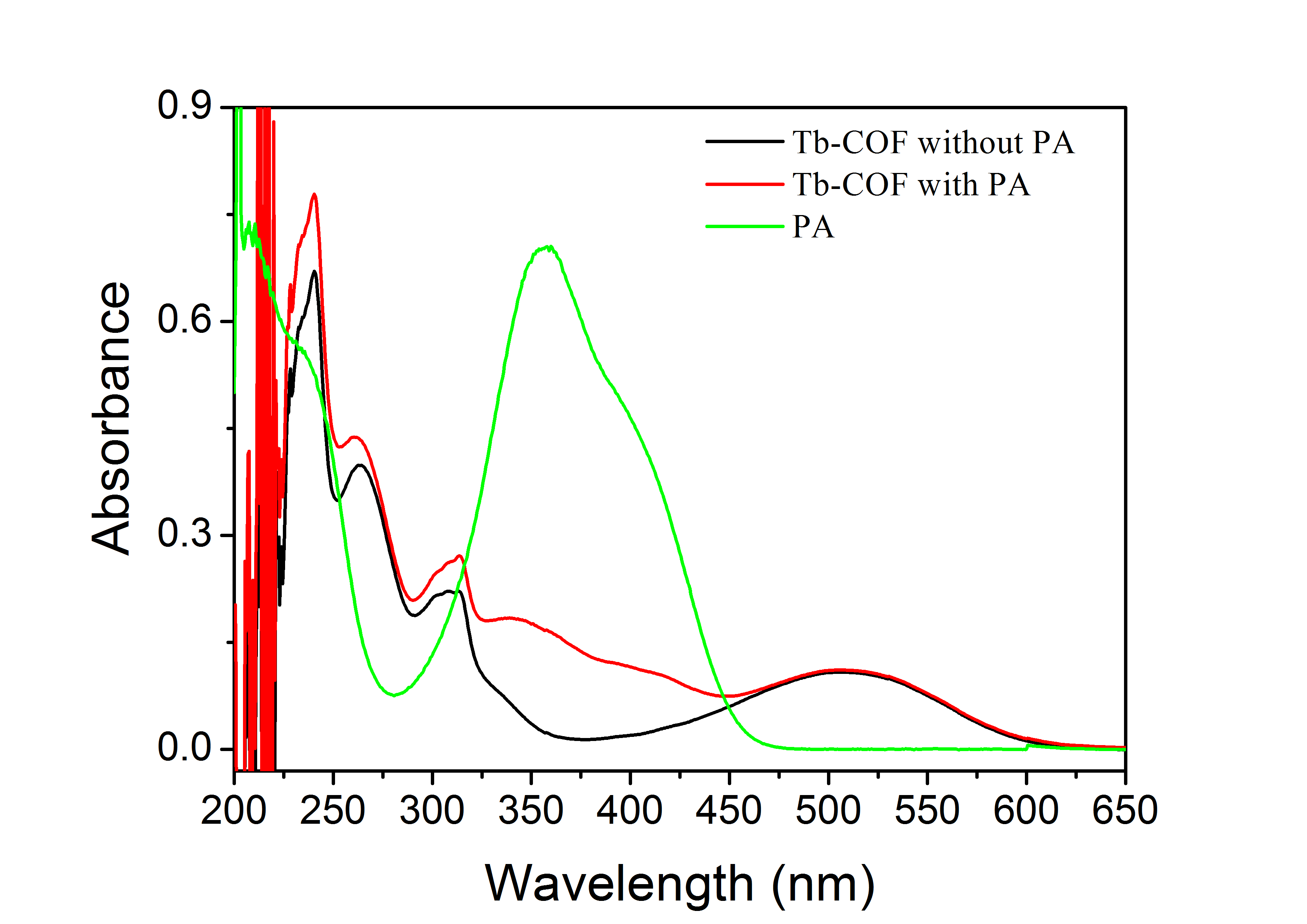

Supplement: Supplementary file 1 [file DataSheet1.docx]
